# Insights into Flow and Continuous Systems in Pharmaceutical Manufacturing: Challenges and Opportunities

**DOI:** 10.1021/acs.oprd.5c00309

**Published:** 2026-02-23

**Authors:** Adrian M. Daly, Seán Hardiman, David Hogan, Gary Morris, Robert Moylan, Barry O’Donovan, Eoin Power, Paul Phillips, Shane Robinson, Philippe M.C. Roth, Megan Smyth, Edmond Magner, Aisling Arthur, Gerard P. McGlacken, Marcus Baumann, Anita R. Maguire

**Affiliations:** † Pfizer Ringaskiddy, Co. Cork P43 X336, Ireland; ‡ Arran Chemical Company, Monksland Industrial Estate, Monksland, Athlone, Co. Roscommon N37 DN24, Ireland; § APC Ltd., Building 11, Cherrywood Business Park, Loughlinstown, Co. Dublin D18 DH50, Ireland; ∥ 1539 Eli Lilly, Dunderrow, Kinsale, Co. Cork P17 NY71, Ireland; ⊥ 697011 SK Biotek, Swords Watery Lane, Swords, Co. Dublin K67 AY91, Ireland; # 201477 MSD Ballydine, Ballydine, Kilsheelan Clonmel, Co. Tipperary, E91 V091, Ireland; ∇ J&J Innovative Medicines Little Island Industrial Estate, Little Island, Co. Cork T45 P663, Ireland; ○ Willy A. Bachofen AG Switzerland, Junkermattstrasse 11, Postfach 944, Muttenz 1 CH-4132, Switzerland; ◆ Almac Sciences, 35887 Almac House, 20 Seagoe Industrial Estate, Craigavon BT63 5QD, Northern Ireland; ¶ Department of Chemical Sciences, Bernal Institute, 8808 University of Limerick, Limerick V94 T9PX, Ireland; ▶ SSPC, The Research Ireland Centre for Pharmaceuticals Bernal Institute, 8795 University of Limerick, Limerick V94 T9PX, Ireland; ▷ School of Chemistry, 8797 University College Cork, College Road, Cork T12 YN60, Ireland; ◀ School of Chemistry, University College Dublin, Bellfield, Dublin 4 D04 N2E2, Ireland; ◁ School of Pharmacy, University College Cork, College Road, Cork T12 YN60, Ireland; 15 SSPC, The Research Ireland Centre for Pharmaceuticals, https://sspc.ie/

**Keywords:** continuous flow, API synthesis, Irish pharmaceutical landscape

## Abstract

Growth and development of the pharmaceutical sector in Ireland has been remarkable over a 50 year period starting as an API manufacturing hub with strong regulatory confidence gradually expanding to include a range of activities ranging from process R&D, formulation services, and other high-value elements of the pharmaceutical enterprise, while in parallel complementary expertise in bioprocessing and biotechnology has resulted in the sector being globally significant and integrated into global supply chains. The more significant change in API manufacture in recent years has been the introduction of continuous flow technology offering many advantages including safety, efficiency, and flexibility. Nevertheless, in such a highly regulated sector navigating the transformation from traditional large-scale batch manufacture to embedding flow technology is not trivial. The Irish landscape, comprising leading multinational API-producing sites, complemented by Contract Development and Manufacturing Organizations (CDMOs) and indigenous companies providing tailored services, has very successfully embraced embedding continuous flow technology for API and RSM manufacture at scale in partnership with the regulatory agencies. In this context, some aspects underpinning this transition are discussed as an informative commentary on the state-of-the-art for the use of flow in API/RSM manufacture.

## Graphical abstract

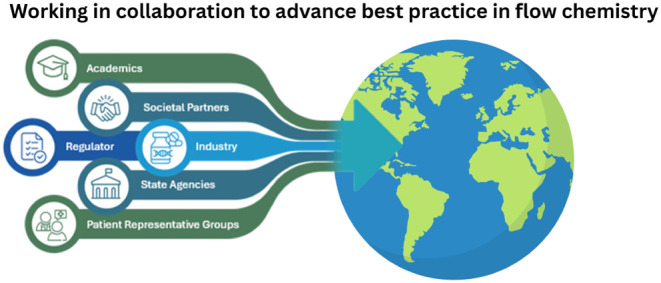

## Context

Ireland plays a vital role in global supply chains within the pharmaceutical and biopharmaceutical sectors with an excellent track record in active pharmaceutical ingredient (API) manufacturing over more than 50 years.^1^ The sector employs over 50,000 people directly, with 61% growth since 2016 reflecting the nation’s strong support for global market demands. Key elements underpinning this success include:

- Availability of a skilled workforce with strong communication and problem-solving skills
- Cluster effect resulting in increased capability at a sector level
- Credibility with the regulators in terms of high-quality operations and products
- Attractive operating environment
- Partnership—across the sector, with academia and policymakers

A multi-institutional research center, SSPC, the Research Ireland Centre for Pharmaceuticals,^2^ had an important impact in bringing together all of the higher education institutions (HEIs) nationally with research expertise relevant to the pharma/biopharma sectors and the industry sector to build lasting relationships and collaborations.^3^ In recent years, one of the strategic research strengths of the Centre has been in the area of Continuous Flow given the growing importance of this in API manufacturing. One of the key goals of SSPC is to ensure a supply of PhD graduates skilled in flow chemistry to drive growth in this area within the pharma sector. At this point, there are a number of internationally recognized PIs in this area within SSPC, contributing to the international visibility of the Centre.

Flow chemistry is particularly attractive in the pharmaceutical industry due to its ability to perform continuous reactions, which can lead to faster, safer, and more environmentally friendly processes compared with traditional batch methods. This technology is gaining momentum, as companies seek to improve their manufacturing capabilities and reduce costs.

In recent years the activity in continuous flow within the pharma sector in Ireland has grown rapidly with large multinational companies leading the way in delivering API processes at scale matched by complementary expertise in indigenous companies.^4,5^ Many of the Irish API sites have played leading roles in the adoption of flow technology and in engaging with the regulators on how flow processes are validated and approved. Within SSPC, a Community of Practice in Flow has been established bringing together thought leaders in this area from both the industry and academic sectors to capture the current status in terms of bringing a continuous flow process through the regulatory approval process and perhaps pointing the way in terms of future developments and solutions to current challenges. The collegial partnership across big pharma, indigenous SMEs, and the academic sector within Ireland is particularly valuable and offers unique opportunities for novel mechanisms of engagements.

### Customers and Historic Vision of Flow Chemistry

Flow chemistry has been part and parcel of the world of chemistry for a long time. For much of the 20th century, flow chemistry was primarily utilized in large-scale industrial applications such as the Haber–Bosch process for ammonia synthesis, which was developed in 1913.^6^ Concomitant with the development of microfluidic systems, interest in flow chemistry began to develop significantly in the early 21st century. The rise of flow chemistry and its use have followed the typical pattern of new synthetic methods (i.e., photochemistry, electrochemistry, and mechanochemistry). More specifically, when microfluidic systems became available at the turn of the millennium, the primary focus in academic research was in process optimization and discovery, using available materials and based on expanding the boundaries of established reactions.^7–11^ Initially a niche topic adopted by some industries and academic groups, after about a decade, the area experienced a resurgence. Research papers attracted much more attention, and the availability of off-the-shelf flow equipment facilitated the translation of batch chemistry to flow, leading to a great increase in the volume of published research on flow chemistry. While some papers offered an advantage over analogous batch methods, this was not necessarily the case in all instances. As the field of flow chemistry matured and became accepted as a viable method for adoption, efforts focused on the key advantages that flow chemistry bestows; moving from a simple batch alternative to an enabling technology to a discovery platform. Today, flow chemistry is a vibrant area of research and application. It is widely used in many synthetic fields such as the pharmaceutical industry for the synthesis of APIs and is gaining traction in other areas of chemical manufacturing.^12–16^ Flow chemistry’s journey from industrial roots to a widely accepted scientific discipline highlights the importance of innovation and collaboration in advancing chemical sciences.

This growth has led to powerful industrial units by different providers:^17^

- Operations running at an industrial scale for 8000+ hours per year, including running in parallel.
- Solid waste reduction of 75%.
- Increased product yield.
- Plant footprint size reduction by up to 90%.

Collaboration between industries and academia has led to the creation of specialized laboratories (including RCPE,^18,19^ CITOS,^20,21^ CSIRO,^22,23^ and others) in developing methods and thus helping any reluctant scientists in the industry to gain confidence in flow. The outcome of such operations often produces a wide range of papers addressing the transition from lab to industrial scale, and commercial initiatives to support companies going through the process such as Snapdragon (now part of Cambrex).^24^

Similarly, researchers from Irish universities have published on flow-based collaborations with industrial partners across Ireland for several years.^25–28^ This has not only led to developing and transferring of new knowledge across the academia-industry boundary but also more importantly has allowed the training of many postgraduate researchers who have found employment in the industry sector since. Critical funding has been provided through SFI/Research Ireland funded centers such as SSPC,^24–26^ IRC Enterprise Partnership Schemes, or directly from industry. The large number of publications in peer-reviewed journals is a testament to the success of these efforts. Unsurprisingly, these outputs include all major companies such as Pfizer,^29–31^ MSD,^32–36^ Lilly,^37–40^ Almac,^41–44^ Corning,^45^ APC,^46^ and J&J Innovative Medicines^47^ showcasing the value of this approach. Global engagement with the flow community is evident.^48,49^

## Pathways to Regulatory Approval

Interest in continuous technology in the pharmaceutical industry has grown significantly over the past decade. Many companies recognize its potential, investing heavily in support of continuous manufacturing technology. However, few continuous processes have been registered successfully, partly due to a lack of specific regulatory guidance. In response to this, agencies like the FDA, EMA, and PMDA affirm that continuous manufacturing fits within existing frameworks, such as ICH Q8, Q9, and Q10. The ICH has also published ICH Q13, providing specific guidance for continuous manufacturing.

Continuous manufacturing presents unique challenges including process control, data management, material tracking, and equipment design for both solution-phase chemistry and solid processes. The process design space—defined by input variables and process parameters—is vital for ensuring quality. Managing continuously operating processes with large numbers of input variables requires understanding multivariate dependencies and establishing robust design space limits through comprehensive experiments.

Key elements specific to continuous manufacturing include:

- Start-up, shutdown, pause, and restart procedures
- Material collection and diversion strategies
- Measurement and control of process parameters from multiple steps running simultaneously
- Fouling

Operational control strategies, such as PAT, automated responses, and online models for material tracking, are essential for managing complex design spaces. Clear documentation and implementation of these strategies are crucial for regulatory approval and ensuring a robust, repeatable process.

The following sections delve into the industrialization of continuous processes. This includes strategies for scaling up, optimizing operational efficiency, and integrating continuous processes into existing manufacturing frameworks. The focus will be on practical approaches and case studies that demonstrate successful implementation in the industry.

### Process Characterization and Control Strategy Development

Devising a control strategy for the manufacture of a drug substance is a key activity in the filing and bringing of a new drug to the market. The control strategy derives from knowledge gained on the process parameters that impact the critical quality attributes (CQAs) of a drug substance and outlines the operating policies and in-process controls (IPCs) that are designed to prevent these CQA-affecting process parameters from deviating outside of an established design space where product quality has been proven.^50,51^ Pharmaceutical companies are required to embark on what is termed a quality-by-design (QbD) approach or process characterization (PC) exercise, which, at its core, encompasses a series of statistically designed experiments to elucidate proven acceptable ranges (PARs) for process parameters within a design space for the synthesis of a drug substance from its registered starting materials (RSMs), in conjunction with other critical tests and knowledge-gathering activities.^52,53^ A preceding process risk assessment provides the foundation to this experimental plan, where process parameters and ranges that are expected to cause variability in the CQAs are nominated and an analysis is undertaken to structure a design of experiments (DoE) that covers the expanse of this design space.^54^

#### Process Characterization of Flow Chemistry

For the batch production of synthetic drug substances and intermediates, the PC practices that most companies follow are time-tested and well-established, following decades of industry focus on developing batch synthetic processes for tech transfer into a batch plant infrastructure that has not varied greatly in its design. Despite the depth of research and interest in continuous manufacturing (CM) of pharmaceuticals over the past 20 years,^55^ this alternative mode of manufacturing remains relatively new, and the industry is still finding its feet with how to navigate PC for integrated continuous processes. Relative to the simplicity of batch manufacturing, where steps and unit operations are often detached from one another, flowing the output from preceding continuous operations or steps directly into the next creates a more complicated multivariate process parameter landscape with a high degree of interdependency between the adjoining unit operations and steps. Consequently, this increases the number of process parameters that need to be considered in the PC exercise for the integrated continuous processes.

Adding to this complication is the requirement to execute PC studies on processing equipment that is representative of the manufacturing scale. A downscaled replica of the envisaged integrated continuous process is typically established in the laboratory at a lower vessel volume for this purpose. However, the high productivity of these systems presents a drawback for implementing PC, as experimentation is material-intensive, time-consuming, and laborious. As such, a modification in approach is required for implementing PC on integrated continuous processes versus batch, one that allows for capturing the critical interdependencies in this expanded multivariate design space in the most material- and time-efficient way possible.

#### Use of Integrated Modeling to Streamline PC

Mathematical modeling has been used to support efficient PC completion on integrated CM processes. In this approach, data from PAR-setting experiments is complemented by a digital evaluation of the multivariate process parameter landscape to provide maximum information for control strategy development with a reduced laboratory effort.^56^ By the development of mechanistic models of the unit operations involved in the process, such as chemical transformations, workup operations, and crystallizations, it is possible to bring these models together in an integrated system model of the process. Such holistic models enable end-to-end simulation of the process, providing a means to explore the impact of process parameter variations and disturbances on CQAs (e.g., residual substrate, purity, and crystal form) and operational performance metrics (e.g., yield, throughput, and process mass intensity) in silico.

Batch experimentation provides a material-efficient option for gaining a first mechanistic understanding of the unit operations involved in the CM train, and commonly batch studies are leveraged to acquire time-series data sets for estimating the parameters of continuous process models. Mechanistic models for application in PC and control strategy design have been developed for a wide variety of chemical transformations occurring in continuous stirred tank reactors (CSTRs) and plug flow reactors (PFRs), where the parameter estimation to determine some of the unknown coefficients in the reaction model was completed by fitting the model to data acquired from batch experiments.^57–61^ Mass transfer, partition, and heat transfer coefficients and thermodynamic properties that are required for simulating continuous workup operations such as liquid–liquid extractions and distillations can also be readily obtained from batch studies.^62^ Combinations of continuous experiments,^63^ and continuous and batch experiments,^64^ have been undertaken to determine the crystal growth and nucleation kinetic expressions needed for population balance equation (PBE) models of continuous crystallization processes to support design space exploration and control strategy establishment for manufacturing processes. When powders need to be isolated at the end of an integrated continuous crystallization process, the operating mode typically reverts to a batch-like process, with approaches that use parallel filter devices to perform semicontinuous filtration, washing, drying, and cake dissolution operations as required, which are common in production-scale demonstrations of integrated CM. In this case, well-known filter models that are based on Darcy’s law^65^ can be modified to account for the semicontinuous nature of the operation and allow for simulation as part of an integrated system model, with appropriate batch-style experiments facilitating parameter estimation.^63^

Integrated system models have several valuable ways in which they can interact with the PC workflow to drive efficiency by bringing a mechanistic perspective to the experimental design. Conducting robust PC for integrated continuous processes necessitates implementing DoE-style experimentation on top of one-factor-at-a-time (OFAT) studies to assess the impact of cross-variations occurring in the parameter space due to a change in a single variable.^56^ In a mixed suspension, mixed product removal (MSMPR) crystallizer, for example, an adjustment in antisolvent feed rate would simultaneously change the antisolvent-to-solute ratio, residence time, and crystallizer mixing characteristics (e.g., mesomixing).^66^ However, given the numerous process parameters present in integrated continuous processes, exploring all possible interactions experimentally would be challenging and labor- and material-intensive. An integrated system model greatly streamlines this effort by enabling a digital investigation of this multivariate parameter space to determine worst-case variable combinations, which can then be prioritized for evaluation in experimental testing.^56,60,62,63^ A further application strength of integrated system models is their utility in digitally assessing continuous processing failure modes that may occur in production such as pump power failure, reactor temperature loss, or transfer line blockage. These evaluations can inform key parts of the control strategy such as recovery protocols that describe how to handle events like these if they were to occur during manufacturing and permissible timeframes for operating out of a state-of-control.

From an in silico failure mode analysis on a blocking risk with a continuous amide coupling reaction that employed a suspension reagent feed of N,N′-carbonyldiimidazole (CDI) in 2-methyltetrahydrofuran (MeTHF), Içten et al.^62^ demonstrated that blockages needed to be resolved within 30 min, with such a blocking event encountered and remedied within 22 min during the production run, proving the procedural control to be effective. The integrated system model used in this example also informed additional elements of the control strategy for this synthetic stage such as proposing the raising of CSTR temperature as a corrective lever for increasing reaction conversion in the event of operational disturbances and transitioning away from a semibatch to a continuous packed bed distillation to improve the robustness of synthetic intermediate product quality by reducing the sensitivity of the distillation performance to upstream parameter fluctuations. Griffin et al.^56^ similarly used an in silico process disturbance analysis to identify permissible time periods for feed pump and vessel temperature power losses, incorporating these model-advised timeframes into PC experiments to verify and assign a tolerable intermittent disturbance time around certain process parameters.

In the postapproval space, a validated integrated system model also retains and extends its value past development and PC and into supporting continual process verification and improvement of the commercial process. Diab et al.^67^ performed a global sensitivity analysis (GSA) with a validated integrated process model that described a commercial multistep CM process. The GSA identified parameters that could be changed within the confines of the regulatory filing to improve the yield.

These case studies highlight the value of incorporating mathematical models into the PC and control strategy development workflow for integrated continuous processes. They demonstrate how a model-supported PC workflow saves time, material, and labor, which are essential factors to consider when pursuing continuous process development as a far greater demand is placed on these resources in CM compared to traditional batch processing.

#### Requirements for Lab-Scale Setups in PC

Although system-level mathematical models greatly expedite and reduce the volume of experiments required in PC, they do not obviate the need to establish a representative scale-down version of the production-scale continuous process for use in PC studies. This lab-scale continuous system needs to be designed in such a way that it faithfully replicates the integration of all unit operations and material transfers from starting materials to powder isolation, while being appropriately scaled down so that material needs are not prohibitive. With an appropriate lab-scale setup, the continuous process can be characterized, assessing aspects such as start-up transition to state-of-control operation, long-term runs for operational sustainability, process parameter variation, shutdown and restart, and disturbance, deviation, and recovery procedures. Critical in the design of the lab-scale continuous system is that the mixing and heat transfer relationships between it and the manufacturing-scale process are known and well understood such that the PC results can be related to the production process.

#### Critical Scaling Parameters

Achieving mixing similarity between production- and lab-scale continuous equipment is complicated by the inability to hold all mixing parameters consistent between the scales.^68^ Factors such as mean residence time (τ_m_), residence time distribution (RTD), axial dispersion number (ADN), Reynolds number (*Re*), energy dissipation (*ε*), mixing time scales (τ_macro_, τ_meso_, and τ_micro_), power number (*P*), geometric similarity, and combinations thereof have been used as scaling parameters for continuous reactors.^68–72^ Selecting the correct mixing parameters to prioritize is an application-specific task that requires a good understanding of the underlying mixing sensitivities in the process. Experiments can be conducted to characterize the effect of mixing parameter change on process performance and elucidate the most important factors to keep consistent throughout scale-up. In the context of PC, these experiments can subsequently permit the establishment of PARs around the mixing parameters that have a significant impact on process performance, which the scaled-up version of the process should be positioned within.

Keeping a consistent τ_m_ during scale-up is logical due to the controlling influence of this parameter on reaction conversion and impurity profile at the exit of PFRs and CSTRs. τ_m_ is calculated by dividing the reactor volume (*V*) by the total volumetric flow rate through the reactor (*Q*) and guides production scale-up based on the straightforward principle of keeping *V*/*Q* constant or within an established PAR. RTD measures how long materials (e.g., reagents, products, and particulates) spend inside a reactor or crystallizer and provides insights into how real-world PFRs, CSTRs, and MSMPR crystallizers deviate from ideal flow behavior due to axial dispersion and imperfect mixing. Scaling flow reactors based on maintaining a consistent Reynolds number flow regime between bench- and production-scale processes is a strategy that is commonly adopted in pharmaceutical applications. In scaling a kinetically fast aldol reaction from a lab- to a production-scale PFR, McMullen et al.^72^ used the relationship between substrate conversion and Reynolds number to devise a scale-up strategy that maintained a transitional flow regime between the reactor scales, enabling consistent reaction performance despite differences in mixer designs and flow reactor sizes.

#### RTD in Homogeneous Systems

Differences in PFR axial dispersion and RTD may arise during scale-up due to variation in the dimensions of the production-scale PFR relative to the lab-scale setup. For instance, considering the length-to-diameter ratio (*L*/*d*) in open-tube PFRs, if flow is in the laminar regime and the objective is to maintain low axial dispersion, then a higher *L*/*d* may be required in the production-scale reactor to achieve this.^73^ Maintaining the RTD in CSTRs is similarly key for preserving reaction performance as the system is scaled, which relies on having consistent mixing efficiency, flow uniformity, and τ_m_ to prevent underreaction, overreaction, and side-product formation as a result of RTD disparity. The determination of RTDs for homogeneous reaction systems using single-phase tracer experiments is straightforward.^56,73^

#### RTD in Heterogeneous Systems

Heterogeneous systems (e.g., biphasic CSTRs and MSMPR crystallizers) present additional RTD management complexities, where the dispersed solid phase in the stirred volume also needs to be well-mixed, in conjunction with suspension transfers between vessels being uniform and representative of the contents of the stirred volume. To adequately describe systems involving an additional solid phase, it is necessary to conduct both liquid and solid tracer experiments to quantify the respective liquid and solid phase RTDs, both of which can be affected by the modified tank hydrodynamics due to the presence of solids. Guo and Yang^74^ performed tracer experiments on the liquid and solid phases of a CSTR test system demonstrating that while the mixing of the liquid phase was close to ideal, the mixing of the solid phase differed significantly, with the outlet pipe position in the bulk also affecting the RTDs of both phases.

Ensuring representative withdrawal from continuous stirred tanks that involve suspensions is critical for preventing preferential vessel deliquoring, particle size classification, and unpredictable operation. This has been a major focus in pharmaceutical continuous crystallization research, with several techniques developed to facilitate fast periodic withdrawal of representative suspensions from small-volume systems that operate at low continuous pump speeds,^71,75–79^ along with methods to test that the system design delivers representative product.^80–83^ These intermittent material transfer methods have wide-ranging utility in an integrated CM train for handling suspension processing operations,^84^ and the technique can be harnessed to enable heterogeneous reactions in CSTRs that involve a solid reagent or precipitate solids.^85^

Research on the full characterization of the solid and liquid phase RTDs in particulate-involving continuous stirred tanks is seldom reported. Several studies have used single-phase tracer experiments to approximate the RTD in such heterogeneous systems, forgoing any explicit evaluation on the effect of solids involvement.^86–89^ Although helpful for setting initial configuration set points that encourage good RTD performance, omitting the solid phase in the RTD characterization can overlook the need for additional refinement of the configuration to mitigate issues related to imperfect solid–liquid mixing. Sha and Palosaari^90^ highlighted that significant stratification in suspension density can occur depending on mixing intensity in continuous MSMPR crystallizers. Pomberger et al.^91^ characterized the solid RTD in a biphasic CSTR cascade, with the results confirming near-ideal CSTR behavior, giving the authors confidence that accumulation and local excess of solids would be avoided in the chemistry run. Recently, Aprile et al.^92^ determined the solid phase RTD of a novel vertical tower continuous crystallization system, which confirmed that particles in the coarse size range experienced nonideal mixing, suggesting that further mixing optimization was needed for representative suspension transport.

These case studies on solid phase RTD evaluations in CSTR and MSMPR crystallizer systems illustrate the numerous pitfalls surrounding the appropriate design of continuous stirred tank systems to facilitate satisfactory two-phase mixing and compositionally representative suspension flow in and out of the vessel. Opportunity exists for greater attention to be given to this topic from the perspectives of: (1) demonstrating the implementation of methods to characterize solid phase RTDs for broader reaction and crystallization use cases; (2) developing general recommendations for optimal system design and scale-up; and (3) publication of reports on the incorporation of solid phase RTD and suspension flow integrity characterization within the pharmaceutical PC workflow. By combining high-resolution hydrodynamic modeling tools with mechanistic models that predict how liquid and solid phases evolve in these systems, potentially the experimental component of this challenging characterization work can be minimized.^83^

#### Control Strategy Considerations in CM

In the context of integrated continuous processes, RTD models play a crucial role in helping define the control strategy for manufacturing. System-level RTD models allow simulation of how materials propagate from feed tanks all the way through interconnected continuous unit operations and into the final drug substance. For material traceability, this provides a framework to mathematically relate raw starting materials to feed solution lots and product containers, which is essential for regulatory compliance. During a CM campaign, feed tanks may be switched over several times with empty tanks resupplied with fresh feed solutions to keep the process running smoothly. RTD models enable the provision of a lot genealogy for the source materials that have gone into making up a given container of a drug substance produced from a CM process. From a process control perspective, system-level RTD models allow for numerous “what-if” material propagation scenarios to be simulated, which can help with defining surge capacity in the integrated continuous process train and material-efficient policies for diverting contaminated materials to waste.^73^

Although RTD models can be integrated with mechanistic models to provide a high-resolution picture of material composition change throughout a unit operation or full CM train, industrial use cases have demonstrated the adoption of simplified system-level RTD models that have been implemented with several considerations that do not necessitate incorporating mechanistic models into the analysis. Cole et al.^93^ reported on the utility of one such simplified system-level RTD model for a CM train of five integrated continuous unit operations, where simulations highlighting the extensive clearance time of an impurity from the train aided decision-making to temporarily pause production when elevated levels of this impurity were observed during a CM campaign. In this case, system-level RTD and heat transfer models were fitted to experimental data generated during a manufacturing-scale demonstration of the synthetic process conducted in development laboratories, using equipment of equivalent size and throughput to the current good manufacturing practices (cGMP) equipment train. Generally, it is recommended to measure τ_m_ and RTD from a run of the actual chemistry at manufacturing conditions, as differences in the fluid properties between room temperature solvent tracer references and the genuine reaction mixture can be significant.^73^ In addition to providing a means for tuning mathematical models to an accurate version of the manufacturing process, the authors noted that the full-scale trial run advantageously allowed for any scale-sensitive issues to be identified and ironed out prior to cGMP manufacturing, increasing the likelihood of a successful tech transfer.^93^

The aforementioned case study highlights a CM control strategy that places emphasis on monitoring the chemical composition of process streams to ensure that the continuous process is operating within a defined state-of-control. An alternative approach is to rely on parametric process control, where quality is assured by monitoring and controlling operating parameters within a set of established PARs that have demonstrated product quality robustness. Griffin et al.^56^ described the implementation of a strict parametric control strategy with discrete lot release for a production-scale integrated continuous process that was underpinned by a rigorous PC effort and eliminated the requirement for any IPCs during manufacturing. The proven robustness of the process from PC provided relatively wide PARs that process parameters could move within, without constituting a deviation requiring diversion of material to waste. The authors noted significant operational benefits from the reduced sampling and testing burden placed on human resources as well as the limited material lost from minimal diversions to waste. While this control strategy necessitates a considerable upfront effort in the PC space to justify the elimination of IPCs in manufacturing, it is supportive of a leaner human footprint on the manufacturing floor, which is efficient and more sustainable for long-term manufacturing compared with approaches that rely heavily on IPCs and round-the-clock quality control (QC) staff and lab involvement.

## Challenges to Develop Technologies to Meet the Requirements in Industry

### Develop Strategic Business Case vs Product-Driven Capital: Equipment Lead Time and Knowledge Build

Small molecule commercial API manufacturing facilities in Ireland have traditionally invested in batch manufacturing rather than continuous manufacturing plant, and the availability of plants to produce commercial supplies of API in continuous mode typically requires investment in technically complex equipment and infrastructure. The scale of investment depends on the strategy adopted by the company, and there are broadly two alternative approaches for pharmaceutical companies to invest in continuous manufacturing:

1. Invest strategically in the necessary technology and infrastructure to enable a technology platform capable of producing a range of manufacturing processes in continuous mode. Such an investment would typically be substantial (either a new building or a substantial investment within an existing building). While such an investment would typically be undertaken based on a view of the development product portfolio, there is a certain level of risk when the high product attrition rate of development pharmaceutical products is considered. On the other hand, the availability of such a facility at a manufacturing site in Ireland creates a huge strategic advantage for the manufacturing site in terms of the allocation of new products to the site in the future. Grant aid, mainly through the Irish Development Authority (IDA), has been very beneficial to this type of strategic investment.

Sites equipped with this level of flexibility also benefit from the agility of being able to quickly react to changes in the demand for an array of products. The capabilities and expertise for the development and manufacturing of API in continuous mode are not readily available in Ireland and investments in continuous manufacturing platforms need to go hand-in-hand with building the knowledge base in continuous manufacturing. In this regard, an increased emphasis on continuous API manufacturing is needed in relevant third-level engineering and science programs.

2. Product-specific investment, meaning that the company invests when a product in the development phase specifically requires continuous manufacturing technology. Such investments are smaller in scale and are tailored toward the specific requirements of the manufacturing process in question. The risk with this approach is that the lead time for the capital project is likely to be rate-limiting for the product launch, and this can necessitate a much smaller scale of project. Cost reduction in the hydrogenation of 2,4-dinitrotoluene was estimated at 37–75% when conducted in flow relative to batch conditions.^94^

### Different Types of Flow Unit Operations/Next Generation of Continuous

As well as deciding whether to complete a strategic upfront investment or a product-driven investment, a company must also determine how to proceed with its design.

1. Examples can include:

- Off-the-shelf designs
- Internal designs
- Joint development with an external SME

Off-the-shelf designs offer the advantage of tried-and-tested designs and equipment and more reliable cost estimates. However, it requires processes to be developed to meet commercial equipment design.

If the required flexibility, scale, or compliance requirements do not exist commercially, then opting for internally designed skids can be advantageous. Such equipment could be constructed internally or via an external skid builder.

If the technology does not exist commercially, then a joint development agreement with a commercial SME to develop and build the required technology can accelerate the development and realization. In the development of new technology, the characterization of heat exchange at different scales is important to ensure processes are safe and scalable.

2. ATEX (ATmospheres EXplosibles refers to the hazard of explosive atmospheres) or non-ATEX rated equipment: As described above, another fundamental decision to be made in CM/flow technology design is whether to incorporate ATEX or non-ATEX rated equipment. ATEX-rated equipment can be installed locally alongside existing ATEX-rated equipment (e.g., batch reactors) or in a non-ATEX-rated area, which must be separated from ATEX zones. In some cases, non-ATEX-rated equipment may be placed in a fume cupboard to provide safety protections. The equipment may therefore also be laboratory-rated equipment. Note that in both ATEX and non-ATEX-rated scenarios, assuming locations allow, the flow/CM equipment may be supplied from/received into batch vessels located in an ATEX environment.

Items to consider when deciding on whether to install ATEX or non-ATEX rated equipment include:

- New or existing build: Retrofitting an existing facility to take non-ATEX-rated equipment/position close to adjoining batch vessels can be challenging.
- CM vs Hybrid: If operating in a hybrid mode, locating portable ATEX-rated equipment beside ATEX vessels can be advantageous.
- Scale/Cycletime: Operating in a non-ATEX fume cupboard will likely result in small equipment/pipelines. Depending on the required production volumes, small equipment will likely result in reactions being run for an extended period, which can potentially cause blockages over time. If operating in hybrid mode, the flow cycletime may be significantly different to the adjoined batch vessels.
- Safety: Operating in a non-ATEX-rated area may offer reduced safety challenges over ATEX rated equipment.
- Commercially available equipment: ATEX-rated versions of equipment may not exist or may come at a cost premium. Lab-scale equipment is generally more readily available.

3. Portability to Internal/CDMO: Many pharma facilities are multiproduct facilities. In Ireland the landscape is evolving to include increased development and commercialization activities. This can result in product production locations changing over the lifespan of a product. This requires technology transfers of products either internally within a company or to an external CDMO. Flow and CM Technology is relatively new to the Pharma Industry, and therefore, the portability of the technology must be considered when opting for flow/CM processing. It can be difficult to have exact replicas of production equipment from one site/CDMO to another. It is, therefore, critical to understand the key attributes of a technology platform that must be replicated to meet process needs.

## Different Types of Flow Unit Operations/Next Generation of Continuous

The main benefits of continuous manufacturing as opposed to batch manufacturing of APIs are the reduction in the chemical process safety risks, improvements in the purity profile, and a reduction in the lead time to get from raw material to the final API. These and other benefits to continuous manufacturing can be summarized:

- Chemical process safety risk reduction occurs in flow processes because the quantity of reacting mixtures in contact at any point in time is substantially lower than in a batch reactor and therefore processes that are inherently hazardous in batch reactors can be carried out under a much lower level of risk in flow.
- Flow enables a precise window of time at the most productive reaction conditions and minimizes the time at less productive conditions. This leads to an improved purity profile with reduced potential for degradation side-products.
- The reduced lead time arises because flow processes can deliver a steady stream of API, whereas batch processes deliver the API in much larger increments but at a slower rate. Typical lead time for a batch process is in the region of 6–12 months depending on complexity, whereas a fully end-to-end continuous process could deliver the first quantities of API in a matter of weeks. In this context it is also important to distinguish continuous manufacturing processes (whereby the end-to-end process is conducted in flow) from hybrid processes (where flow operations are integrated with traditional batch chemistry unit operations).
- Flow chemistry can exert efficiencies in heating and cooling. Examples of this can include cryogenic reactions such as lithiation chemistry, which can require reaction temperatures of −70 °C and below, which is outside the operating conditions of most industrial chiller ranges. Carrying out this chemistry in flow lowers the cooling duty (the rate at which heat energy must be removed to maintain the desired temperature) and, in most cases, increases the ideal reaction temperatures within a range that is more manageable and economic at scale. Even if under cryogenic conditions, the ability of flow to permit a short reaction time before quenching/reaction allows chemistry to be conducted that would be impossible under batch conditions.
- Flow chemistry can allow for a significantly reduced manufacturing footprint.
- Reduction in the number and scale of reactor cleaning can be enabled by flow, especially with telescoped processes.
- Flow chemistry can also unlock completely new reaction capability that is not available with conventional batch reactions.^95^
- In the future, flow chemistry could potentially allow the production of medicine at remote locations, enabling the delivery of medicines to patients locally in instances of extreme weather conditions or where physical access is difficult.^96^

Flow technology can be applied to most unit operations used traditionally in small molecule API manufacturing processes. Increasingly R&D is being performed on more specialized processes including electrochemical and photochemical processes. Application of flow chemistry as an alternative to batch manufacture may be considered as part of the initial process development prior to the launch and regulatory approval of a new product or as a postapproval change. In both cases a technical justification to verify the superiority of the flow process is required and this can be based on economic (throughput, yield), quality (purity profile) or process safety considerations among others. The overall process may be hybrid, meaning certain unit operations are carried out in batch and others in continuous or it may be fully continuous.

Reaction chemistry is the most common unit operation to which flow chemistry is applied in small molecule API manufacturing, and several technology platforms are available to run these chemistries. The size of these technologies is substantially smaller than traditional batch reactors and a typical setup involves the assembly of the flow reactor on a mobile skid, which can be moved to the necessary location (typically a walk-in fume hood or an ATEX-rated location on the plant floor). Plate reactors are commonly used, and the different reactant streams are introduced into the plate reactor under specific predefined conditions of flow rate, pressure, and temperature. The reaction happens in a continuous manner when these streams come in contact, and this leads to a steady stream of the product solution coming out of the end of the reactor.

Extractions or phase separations are commonly used unit operations after reaction completion, and the purpose of these operations is to purify the product solution. Phase separations take advantage of differences in solubility between the desired product and undesired byproducts as a means of separation. In batch reactors, phase separations are run by agitating the biphasic system for a set time before allowing the layers to settle. Separation is then achieved by transferring the two distinct phases to separate receiver tanks. The same outcome can be achieved in flow, whereby the biphasic mixture is transferred under set conditions to a centrifugal extractor. The centrifugal extractor utilizes the difference in density of the two solvents as a means of separation. Under the developed conditions of flow rate and a sufficient number of revolutions per minute, the two streams are separated in a continuous manner. Furthermore, membrane separators and gravity separators have both been demonstrated at scale and have benefits.

Continuous distillations involve feeding a steady stream into a piece of distillation equipment and achieving a steady state, whereby an equilibrium is maintained between the rate of feed input and the rate of distillate stream output. A good example of such a setup is a wiped film evaporator (WFE), whereby the equipment creates a continuous thin film of solution on the internal wall of the evaporator, from which the volatile solvents are evaporated and removed to distillates. Benefits of such a setup include a significantly reduced thermal stress on the product and a consequent improved purity profile. Another benefit of WFE is the ability to use very low pressures and distill high-boiling materials not typically possible in batch mode.

Crystallization processes in batch reactors are a means of purifying the reaction product by the selective crystallization of the pure product from the crude reaction solution. Crystallization processes are also frequently designed to control physical attributes of the product such as particle size distribution and polymorphic form and therefore can be utilized to tailor the behavior of the pharmaceutical product in the human body. Typically, continuous crystallizations are conducted in a cascade of CSTRs (continuous stirred tank reactors) with aliquots of the crystallization mixture being transferred to the subsequent CSTR at defined intervals. Process conditions (for example, temperature or concentration) within each CSTR in the cascade can be modified so that the crystallization condition of the mixture progresses toward the crystallization end point. Similar to other unit operations in flow, the physical size of the CSTRs (typically a few liters) is much smaller than traditional batch crystallizers (typically a few thousand liters). This offers benefits in terms of the physical footprint of the equipment as well as flexibility and mobility of operation.

The final common unit operation for batch manufacturing processes is product isolation and drying. While there is potential for the implementation of this operation in flow, it is not commonly used. Isolation and drying are typically still done in batch mode.

Experience has shown that automated semicontinuous isolation from a loop allows for the advantages of batch crystallization in terms of control while maximizing efficiency.

### Examples of Chemistry Types Particularly Suited to Flow Chemistry

#### Photochemistry in Flow (Photoflow)

Photoflow is a technology that utilizes the power of photochemistry in flow and has gained significant traction.^10^ Photoflow involves passing a stream of reactants through a clear pipe that is exposed to a light source. The Beer–Lambert law depicts the limitation of the scale-up of the photochemical reaction in batch. However, the implementation of clear (narrow) tubing obviates the inherent limitations of a long path length and opacity. The availability of off-the-shelf photoflow infrastructure is relatively new. However, a small number of SME’s are offering commercial flow/CM units. Equipment designs include plate designs and tubular designs. Items to consider when selecting a photoflow reactor include the scale of operation and whether multiple reactors are needed. If multiple reactors are needed, deciding whether to use parallel reactors or reactors in series will be required. Being able to vary the wavelength of light or utilize LED lights may also be important to the design. The use of continuous flow to access marketed drug products has clearly demonstrated the power of this approach.^97^

#### Immobilized Biocatalysis in Flow

Biocatalysis in flow offers a number of advantages over batch processes. These include enhanced efficiency and scalability, improved quality control, easier purification, and reduced waste.^98^ The setup usually involves a supported enzyme, typically as part of a flow column. When a resin is used as a support, the column is used to retain the resin (aiding resin waste removal). Once the immobilized enzyme bed is loaded on the column, the batch can be passed through the column, where the reaction is catalyzed by the enzyme. Good residence time distribution is achieved via the use of a Dynamic Axial Compression (DAC) chromatography column, which moves a piston along the column’s axis to ensure consistent distribution of the enzyme. Reaction duration can depend on enzyme stability. At the end of the reaction, the column is removed from the reactor. When resin is used, it can be discharged from the base of the column, or depending on its properties, it may be possible to discharge the resin as a slurry (as would be typical in chromatography). The onboarding of biocatalysis in flow by the pharmaceutical industry has not accelerated to the same degree as with other processes, it will continue to offer an excellent process option.^99^

#### Hydrogenation in Flow

The use of flow in hydrogenation reactions offers enormous safety benefits. Hydrogenation can be supplied (and even generated) to the reaction media as needed.^100,101^ Operating a hydrogenation in flow, for example, in a trickle bed reactor, reduces the safety risks associated with a traditional batch hydrogenation, due to the reduction of the scale of the reaction unit, and the amount of reacting batch material at any one time. This area of flow chemistry enjoys increasing interest within the pharmaceutical industry^102^ as companies aim to improve safety standards and move away from traditional high-pressure batch hydrogenation, which has inherent safety liabilities for companies. Hydrogenation remains a very important tool in RSM and API synthesis. Therefore, finding a safer/lower-risk alternative to batch hydrogenation, such as flow hydrogenation, is critical to maintaining the flexibility of API/RSM manufacturers.

## Automation, Modeling, Analytics, and Control

Control and understanding of continuous processes are built upon a robust data architecture combined with the deployment of tools and technologies that are appropriate to the product and/or process lifestyle stages and ultimately deliver a control strategy that is appropriate for commercial GMP manufacture.^103^ This integration allows for real-time monitoring and, if appropriate, adjustment of parameters, such as flow rates, temperatures, and pressures, ensuring optimal performance and product quality. Advanced sensors and automated control systems collect and analyze data continuously, providing feedback that can be used to fine-tune the process dynamically. This approach not only enhances precision and efficiency but also significantly reduces the risk of errors and deviations, leading to more consistent and reliable outcomes. To be able to operate a commercial manufacturing process in this manner, various tools, e.g., “simple” sensors, spectroscopy, online chromatography, data analysis (ML, AI, etc.), and process modeling, can be used throughout the development lifecycle to ensure a robust process with an appropriate control strategy is available.

To maximize value and avoid repeat work it is beneficial to standardize the technologies and hardware and software platforms used. This will maximize the potential for the application of data and models in a seamless manner across all lifecycle stages. The effort to achieve such standardization across R&D and manufacturing sites, typically in multiple different locations, should not be underestimated, even in organizations where all such work is done in-house. In this context, the location of clinical and commercial manufacturing facilities within the one manufacturing site is likely very beneficial.

In Table 1 we describe some considerations across product lifecycle stages for the application and end use of instrumentation, data, modeling, PAT, and process control.

**Table 1:** Automation, Modeling, Analytics, and Control Approaches across Product Lifecycle

Lifecyle Stage			
Element	Process Development	Clinical Manufacture	Commercial Manufacture
Instrumentation	“simple” sensors, in-line spectroscopy, online chromatography on all unit ops	“simple” sensors, in-line spectroscopy, online chromatography on most unit ops	“Simple” sensors, targeted spectroscopy, online chromatography
Data Architecture	Data collection on a unit op/equipment basis	Integrated data collection and analysis	Integrated data collection and analysis
Automation	Minimal automation, focus on flexibility	Automation for consistency of operation	Recipe driven automation for process control
PAT	Collect Data and develop process understanding and identify control requirements or opportunities	Demonstrate required PAT applications and prepare for implementation in commercial manufacture	Targeted application of PAT for specific control strategy requirements.
Process Modeling/Digital Twin (RTD, Kinetics, Dynamics, Genealogy)	Collect data and use appropriate modeling techniques to support process development and optimization	Model evaluation and refinement.	Use of models that are required for the control strategy or can aid management of change
Process Control	Manual control based on real-time observations	Demonstrate control strategy and prepare for commercial manufacture	Automated control of parameters and targeted advanced Control

Many of the considerations in terms of data analysis and automation are shared across both batch and flow processes, including modeling to aid workflow development and prediction of long-term processes, the significance of data collection, analysis, and management. The future evolution of digital enablers, PAT, and AI will inevitably impact significantly over the next decade.

From a CDMO perspective, the drive for automation brings challenges in terms of fast-changing processes during development. Automation and data collection play very important roles in relation to API synthesis through flow chemistry. Data collection and analysis are enablers of development, whereas automation speaks to control of the process as a huge driver for efficiency for both batch and flow processing but may be best suited for commercially stable processes.

## Sustainable Processes and Green Chemistries

The pharmaceutical industry is undergoing a significant transformation toward sustainability, driven by the need to reduce environmental impact and improve efficiency. One of the key areas of focus is the adoption of sustainable processes and green chemistries in the continuous manufacture of Active Pharmaceutical Ingredients (APIs), in line with the well-established *Principles of Green Chemistry*, with particular impact in increasing safety, energy efficiency, and real time analysis to reduce waste.^104^

### Continuous Manufacturing in API Production

Continuous manufacturing can form a key element in the green-chemistry landscape of a pharmaceutical company and may lead to significant improvements in efficiency and sustainability. Some of the benefits of continuous manufacturing include:

- Enhanced Efficiency: Continuous processes can be more efficient than batch processes, as they allow for the constant production of APIs without the need for downtime between batches.
- Reduced Waste: Continuous manufacturing can reduce waste by minimizing the quantity of raw materials and energy required for production.
- Improved Product Quality: Continuous processes can provide more consistent product quality, as they allow for better control over the production process with the potential to reduce the number of the purification steps required to achieve product quality (i.e., less solvent usage and less waste).

A recent publication quantified the impact of flow chemistry from a sustainability perspective with an average 78% reduction in energy consumption, an average E-factor reduction of 87%, 50–90% reduction in water usage and 79% lower CO_2_ emissions compared to batch processes.^105^

### Industry Initiatives and Case Studies

Several pharmaceutical companies have successfully implemented green chemistry principles and continuous manufacturing processes. In recent years, several reviews have been published highlighting Green Chemistry advances associated with transitioning to continuous manufacturing and related case studies.^106–108^

A 2014 Chemtrix Whitepaper—“Continuous Manufacturing – Producing More with Less” highlighted that, as part of the American Chemical Society (ACS) Green Chemistry Institute (GCI) Pharmaceutical Roundtable, Poechlauer and colleagues^109^ were assigned a target to “*develop a business case for continuous manufacturing – compile examples from multiple member companies to demonstrate the green engineering business case”.* They summarized the findings of the workgroup (whose members included representatives from AstraZeneca, Bristol-Myers Squibb, DSM, Eli Lilly, Hoffmann-La Roche and its US subsidiary Genentech, Merck, Novartis, and Pfizer), and identified four business value categories:

- Increased development speed
- Higher process reliability/product quality
- Lower investments in hardware
- Safely scale hazardous chemistry

Interestingly, of the business cases investigated, the Team found that most of the cases fulfilling the above four categories also met many of the Twelve Principles of Green Chemistry, illustrating in this case the significant overlap of green and safe chemistry with company cost targets.

The whitepaper also outlined several company and academic case studies and the benefits of the application of green chemistry in continuous API manufacturing in terms of improving sustainability metrics. By adopting continuous processes and through improvements in process intensification and increased operating efficiency, significant reductions in Process-Mass-Intensity (PMI), Total CO_2_–Release (TCR), energy utilization, and E-factor were realized.

### Challenges and Future Directions

Despite the numerous benefits, there are several challenges associated with the implementation of green chemistry and continuous manufacturing in API production. These challenges include:

- Regulatory Approval: Changes to manufacturing processes may require regulatory approval to ensure the continued safety and efficacy of the drug products.
- Initial Investment: The transition to continuous manufacturing can require significant initial investment in new equipment and infrastructure.
- Technical Expertise: Implementing continuous processes and green chemistry principles requires specialized technical expertise, which may not be readily available within all organizations.

Looking ahead, the pharmaceutical industry will continue to innovate and collaborate to overcome these challenges to achieve more sustainable and efficient production processes, ultimately benefiting both the environment and public health.

## Contract Development and Manufacturing Organization (CDMO)

### A CDMO Perspective of Continuous API/RSM or “Flow” Chemistry

#### How Does the CDMO Landscape Differ?

The pharmaceutical industry has started to embrace the development of end-to-end processes, an elegant and efficient application of continuous flow, often propelled by sustainability measures. This is particularly advantageous when a flow rig is dedicated to the production of a single product (API, Critical Raw Material (CRM), Drug product, etc.), but the situation for those in the Contract Development and Manufacturing Organization (CDMO) space is different.

To stay competitive, CDMOs in Ireland have adopted a strategy where flow rigs are mounted on skids to provide modular, flexible, and adaptable manufacturing solutions that can cater to a wide range of chemistries and can be modified quickly to allow the next contract manufacturing to take place. This supports the financial viability of flow processing investments to ensure an acceptable return on investment (ROI), not by linking the return to a single project but rather by developing a toolbox approach where the modular infrastructure can adapt to the production scale and chemical processing requirements of multiple projects/products. Irish CDMOs have extensive experience in batch synthesis of complex organic molecules, but over the past decade, flow chemistry and continuous manufacturing have become a crucial part of their service offerings.

Although driven by the advantages of continuous flow to access inherently safer processes due to the use of lower reactive volumes, increased temperature control, and access to higher pressure with controlled risk, the key driver for CDMOs has been the access to challenging reactions on scales larger than typically achievable in traditional batch processing. Since the pandemic, there has also been a notable competitive advantage for CDMOs based in Ireland as many corporate social responsibility (CSR) directives have led to a reshoring of CRMs, building blocks, and intermediates to western suppliers. This is further driven by the security of supply from an Irish CDMO (robust, reliable supply chains), excellent quality systems (reliable Good Manufacturing Practice (GMP) and ISO standards), and utilization of technologies to enable hazardous chemistries to be completed.

From a CDMO perspective, flow needs to be assessed early in the product life cycle, and sufficient time is allowed to develop the chemistry. This is often a challenge for accelerated development, where production is outsourced. Flow often initially needs higher quantities of starting materials, which can be a challenge for early development. While a flow reactor will have a small volume, the volume of feed and collection tanks is also a factor.

#### Advantages of Flow for a CDMO Competitiveness

The ability to perform continuous flow can unlock new routes of synthesis not previously viable and can be used to access new increasingly complex molecular entities often in shorter timelines. Figure 1 showcases selected examples of flow processes developed by CDMOs in Ireland.

**Figure 1:**
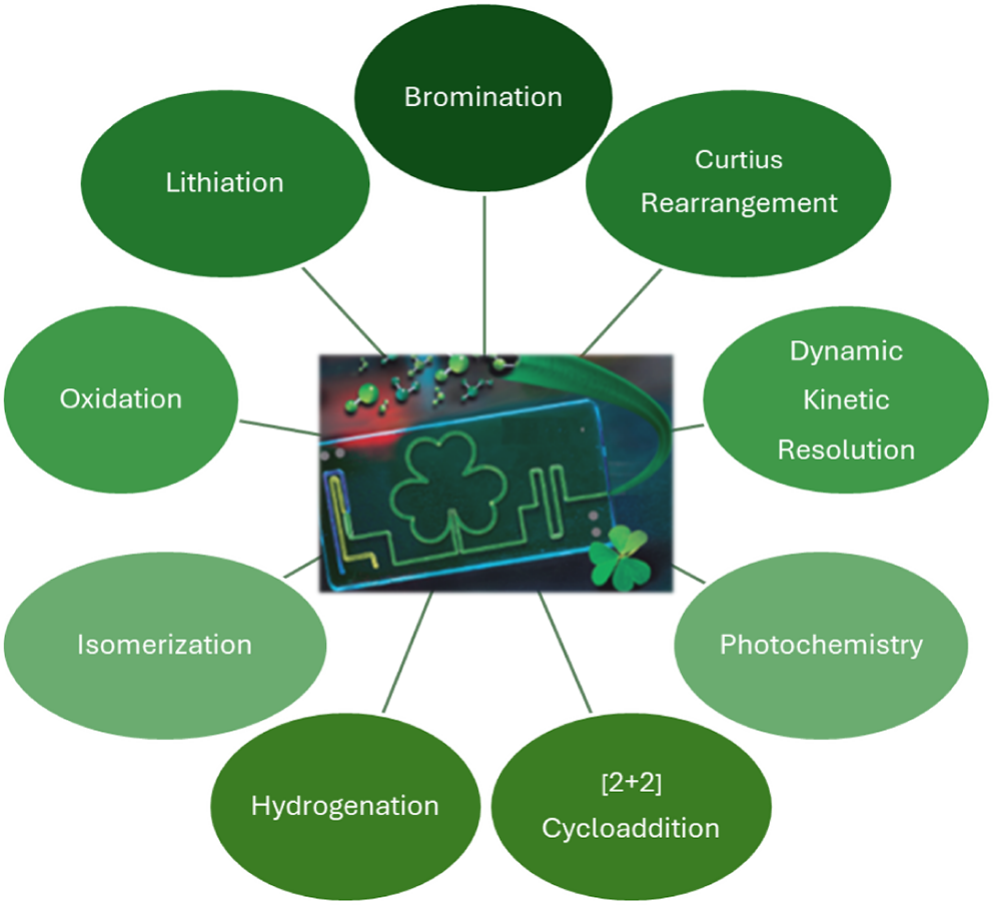
Selected examples of chemistries that have transitioned to flow within CDMOs (graphic reproduced through permission from Almac).

Over the past ten years, the EU-based CDMOs sector has seen a surge in competition from lower-cost countries. As the pressures on the expenses of pharmaceutical development and production escalate in tandem with demand, flow chemistry provides value to organizations through operational cost savings (OPEX). This is achieved by reducing reactor time as flow enables quicker reactions, in addition to offering environmental benefits such as minimizing waste generation and enhancing safety. Importantly, it allows for the exploration of new or highly challenging reactions, especially in situations involving high pressure, high energy, oxidation, or photochemical transformations.

##### Safety

Flow chemistry can allow the scale-up of chemistry that would otherwise be too expensive, too hazardous, and/or too high of a risk from a safety perspective to perform in large-scale batch reactors. For example, the safety and quality benefits of flow Grignard generation are clear.^59,110–112^ The transition to flow has involved significant cultural change and education among chemists, analysts, and chemical engineers.

#### Challenges Moving to Flow Chemistry and Strategies to Address GMP Requirements

For some CDMOs there is concurrent development of processes for both non-GMP and GMP flow manufacturing. This approach has proven advantageous, as insights gained from non-GMP equipment can be utilized to aid in the design and subsequent validation plan of GMP equipment and processes. The initial strategy is to develop processes within development laboratories and tech transfer to non-GMP production equipment. This would allow troubleshooting and flexibility to define parameters prior to “locking in” the process for a GMP manufacture. Alternatively, the utilization of flow chemistry steps at the non-GMP starting materials stage enables greater flexibility prior to the GMP manufacturing steps.

As the industry shifts toward continuous processing, it is crucial that regulatory bodies keep pace with these changes. Ireland holds a strategic edge in this regard, with the Health Products Regulatory Authority (HPRA) actively participating in numerous ongoing flow initiatives. Despite significant progress, challenges persist in areas such as cleaning verification and validation and process control.

### Beyond Batch; The Unique Opportunities of Continuous Flow

A key reaction class that is an obvious contender for continuous flow is low-temperature organometallic synthesis, in particular, lithio species. Reactions of this type are often very exothermic and sensitive and proceed rapidly, which can make them difficult or impossible to control under batch processing. The necessity for cryogenic reaction temperatures to avoid decomposition of the highly unstable lithiated species prior to the electrophilic quench can often be circumvented under continuous flow. This is due to the more efficient heat dissipation from increased surface/volume ratios in the equipment. Remarkably, examples of flow have even been demonstrated at ambient temperature. The fast reactions and capability for an inline quench of the electrophiles facilitate very short residence times, which in turn means high throughputs (kg/day) can be achieved with even lab-scale flow rigs. An example of this type of chemistry being run at scale is shown in Figure 2 by Arran Chemical Company (part of the Almac Group). Many CDMOs may be limited by cryogenic batch vessel size, and utilization of continuous flow as an enabling technology is an excellent option to address the manufacture of CRMs.

**Figure 2:**
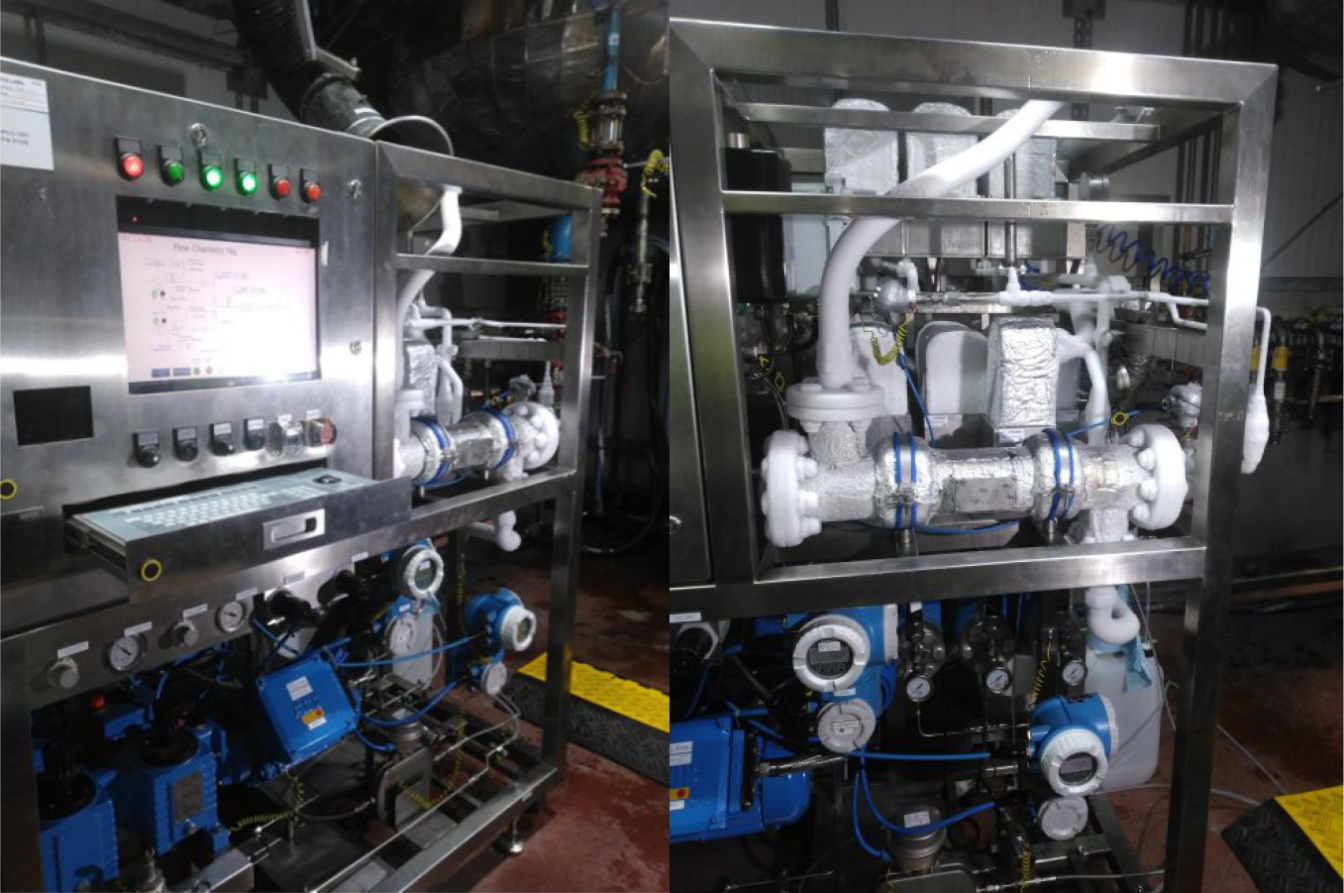
Modular Cryogenic flow reactor skid reproduced with permission of Almac Sciences/Arran Chemical Company.

For a CDMO, material availability for development work during technology transfer can be limited, depending on the stage of development and manufacturing experience. This can be a challenge for familiarization and optimization generally. In recognition of these challenges some CDMOs have been collaborating^113^ to develop specific 3D-printed stainless steel reactors with enhanced mixing efficiencies and the flexibility to redesign and print quickly and cheaply.^114^

## Case Study 1: High-Pressure Hydrogenation in Flow

One key area with potentially wide application for CDMOs would be continuous-flow high-pressure hydrogenations.^100,101^ With capabilities of up to 300 °C and 100 bar pressure using packed-bed reactors and pelletized catalysts, significant expertise for a wide range of transformations such as alkene saturations, nitro group reductions, and reductive deuterations using D_2_ gas can be scaled up to hundreds of kilograms.

One particular case study from Almac involved an alkene saturation where the customer required ∼120 kg of material processed by high-pressure hydrogenation. Accessing the alkene and subsequent steps to the API, required a multistep synthesis. The early steps capitalized on in-house Grignard expertise in batch, and subsequent downstream steps after the alkene saturation exploited a bioreduction technology using carbonyl reductase enzymes. In the reduction step, the initial development work demonstrated that flow reduction produced a superior quality product (GC purity flow ≥ 95.5% vs batch at <90.0%), offered easier workup (no need for Celite filtration) and significant cost savings due to lower Pd requirements (<1.5% catalyst loading vs batch 10%w/w wet loading).

The challenge with the chemistry in batch was that as the scale (2–20 L) increased, the purity decreased. In flow, a robust and reproducible process was developed with a consistent purity profile reported upon upscaling (volume of the packed bed reactor: 6 mL development column vs 78 mL pilot production column). Flow also offered an additional advantage due to the instability of the starting material, meaning it could be produced in smaller batches to be consumed “just-in-time” within the flow rig. Operating under continuous flow with short residence times circumvented the lengthy heat-up and hold times associated with batch hydrogenation, thus enabling enhanced control over the reaction and reduced byproduct formation. Overall, for this process, flow unlocked access to the necessary scale required and future-proofed the process for larger campaigns.

### Platform Technology and Future Flow

An important approach for CDMOs is to pinpoint chemical procedures where flow can act as a facilitator to satisfy stringent processing demands and ensure a reliable future supply. Skids that are modular and flexible offer the greatest adaptability for the successful development of diverse chemistries. As flow chemistry becomes more prevalent, it is expected that continuous flow services at CDMO facilities will revolutionize not only the pharmaceutical sector but also the specialty, agrochemical, and flavor and fragrance (F&F) industries.

## Case Study 2: Continuous Crystallization for Enhancing API Purity and Form Control

Achieving high purity and consistent crystal forms in API manufacturing is essential for ensuring the safety and efficacy of drug products. While batch crystallization has traditionally been the standard technique for separating and purifying APIs, challenges can arise with the control of CQAs as processes scale. Consequently, continuous crystallization has garnered significant attention as a platform method for delivering more reliable CQA control during manufacturing, owing to the ability of continuous technologies to operate within a steady-state regime.

This strategy was leveraged by APC Ltd., in collaboration with a client, to develop a continuous crystallization process for an API designed to treat severe asthma, which provided improved solid-state CQA control compared with an existing two-step batch recrystallization procedure.^79^ The primary driver for this transition was the need for a robust and scalable method to reject a key regioisomer impurity, which had become increasingly difficult to control at the manufacturing scale. A secondary objective was to demonstrate that the desired hydrate form of the API could be continuously crystallized from the solvent system used in the impurity-purging step, thereby confirming that the critical outcomes of the original batch procedure could be achieved in a continuous process.

Initial investigations into the impurity contamination mechanism revealed that during the batch crystallization procedure the solution became supersaturated with the undesired regioisomer. In batch mode, the extended time spent in this supersaturated state allowed the impurity to nucleate and crystallize as a separate solid phase from the API crystals. This understanding motivated a strategy that leveraged a continuous MSMPR crystallizer to operate the crystallization with a residence time that was shorter than the time required for regioisomer nucleation. Under these conditions, the regioisomer remains dissolved at its peak supersaturation and is selectively removed with the mother liquor, preventing its incorporation into the isolated solid product. Batch desupersaturation studies provided the kinetic basis for selecting residence times that mitigate regioisomer nucleation in the continuous crystallization process.

The batch impurity control recrystallization was adapted to a bench-scale continuous MSMPR crystallizer, and a 2.1 h (10.5 volume turnovers) continuous demonstration run was performed to evaluate the effectiveness of the continuous operation in controlling regioisomer rejection. The continuous crystallization achieved a steady-state regioisomer rejection of 97.9%, significantly outperforming the batch process (32.4%). By mass balance, the solid product of the steady-state continuous process was expected to meet manufacturing specifications for regioisomer content in the drug substance, while the batch-produced material was expected to fail this threshold. Due to the short residence time set in the impurity control demonstration run, the MSMPR crystallizer operated at a high API supersaturation, resulting in a lower API yield (72.9%) compared to the batch process (90.5%). Optimization of the residence time was identified as a key focus for future work to balance impurity rejection and yield.

Without water in the solvent matrix, the continuous crystallization process produced the undesired anhydrate form E of the API. Isothermal slurry aging experiments were conducted to identify the optimal thermodynamic conditions for converting form E to the desired hydrate form A. From this understanding, a continuous crystallization run was performed at favorable thermodynamic conditions for form A (>16.6 w% water, 60 °C) to assess whether the required hydrate form of the API could be selectively produced from the MSMPR crystallizer. Under these conditions, the continuous crystallization successfully produced form A, as confirmed by Raman spectroscopy over the course of a 1.8 h (12.3 volume turnovers) continuous demonstration run.

These proof-of-concept trials confirmed that continuous crystallization can effectively replace the two-step batch recrystallization process, successfully meeting both impurity rejection and form control requirements, albeit with a lower recovery. Although further optimization is needed, the continuous process demonstrates significant potential for enhanced impurity rejection, form control, and scalability in API production. This work shows how continuous crystallization offers a pathway to more precise and reliable control of CQAs in drug substance manufacturing.

## Training: Academic-Industry Collaboration to Deliver Interdisciplinary Skills and Expertise

Flow chemistry is here to stay. The basic premises of high surface-to-volume ratios, enhanced mass transfer, smaller footprint, reduced hazard potential, and the portable nature of the reactors will continue to underpin and drive flow technology. Some 20 years ago, a review of reactions in the fine chemical/pharmaceutical industry determined that about 50% of reactions could benefit from continuous flow.^103^ Many of these processes have since transitioned to flow processes, but challenges remain, such as methodologies involving the solid phase, the cost of setup (initially for test cases), lack of widespread availability of equipment, and training.

Successful implementation of continuous flow processing requires access to a skilled workforce familiar with the benefits, opportunities, technologies, and challenges in this area. Chemists, chemical engineers, formulation scientists, data scientists, automation experts, etc., must adjust their approach from traditional batch processing to ensure they deliver effectively in flow processing. However, in addition to ensuring each of these disciplines adapt their curriculum and training to incorporate flow processing, a more fundamental shift toward interdisciplinarity is required. Successful delivery in flow requires interdisciplinary engagement with the science and engineering intrinsically interwoven from the outset. Furthermore, the potential for the use of AI/ML/data analysis in optimization and process understanding is enormous if embedded from the design phase.

Accordingly, a step change in education is required across all of the underpinning disciplines to introduce the concept of flow processing, the benefits, challenges, and most importantly how this differs from traditional batch processing. Inclusion of this into undergraduate curricula perhaps in the area of modern processing technologies, together with one or two lab experiments to illustrate the concept, is critical.

More importantly, the development of advanced skills in this area is required among postgraduate researchers, especially PhD students, and participants on taught postgraduate courses. There is a need to ensure students have a level of understanding of modeling, and insight into the use of approaches such as photochemical and electrochemical methods in flow systems. While individual research teams can drive this to a certain extent, there is a clear benefit from a cohort-based approach in this regard. This approach enables broader access to the infrastructure and collaborative work across interdisciplinary boundaries. Embedding continuous flow processing in structured PhD programs provides PhD students with an introductory-level module so that they have a basic level of knowledge on flow from an engineering perspective. Building on this with advanced-level modules on flow for in-depth research projects is a clear need across all relevant disciplines. Direct involvement from industry offers enormous advantages in terms of ensuring the work within the Higher Education Institutes (HEIs) is in line with the current state of the art in industry and is informed by regulatory requirements in this area. The leadership in the pharmaceutical sector in continuous flow processing and the value of collaboration with HEIs cannot be overstated in this area. Collaborative research initiatives such as SSPC and others (CMAC,^115^ RCPE,^116^ A-STAR,^117^ and CITOS^118^) provide an ideal environment to accelerate skills development in this regard ensuring that PhD graduates are ready to hit the ground running on transition to the industry environment.

There is a very real value to the inclusion of vendors of flow technology in training initiatives given their experience in the design of novel technologies and products to address specific challenges and needs.

Upskilling through continuing education programs is very important in continuous flow processing as many employees in the pharmaceutical sector have had limited or no exposure to this and need to upskill to meet new areas of expertise. Once again, initiatives like SSPC provide an ideal environment for such courses and programs sharing know-how, infrastructure, and knowledge in a safe space and raising the awareness of all, including those providing the training.

As an example, the School of Chemistry at University College Dublin introduced a lecture series on the topic of continuous flow chemistry for academic and industrial applications in 2018 through the appointment of an academic staff member with an active research background in this area. This has led to the delivery of course material for all final year undergraduate students as well as postgraduates (1st year PhD and taught MSc) in parallel to offering research projects in the field of flow chemistry ranging from 6-month final year projects to 4-year PhD projects. More recently, a flow chemistry lab has been introduced at UCD in the third year Chemistry curriculum, where students perform an S_N_Ar reaction under high temperature/pressure conditions in a commercial flow reactor from Vapourtec. This experiment is coupled with a DoE study to combine new aspects (continuous processing, statistical analysis) that are often/commonly missing in chemistry undergraduate laboratories despite their relevance in industrial settings. While the concept of flow is embedded in many chemical engineering degree programs, this usually focuses on the processing of material rather than chemical transformations within flow processing. On the other hand, undergraduate chemistry programs focus on the chemical transformation with limited exposure to flow. Bringing these two isolated approaches together into a coherent interdisciplinary approach is essential–interdisciplinary centers such as SSPC and industry collaboration enrich the student experience in this regard.

From these important initiatives academics are responding to the need to provide chemistry students with much needed training in technology-based synthesis such as flow chemistry, but a major bottleneck is the costly purchase of dedicated flow reactor setups that are ready to use and offer flexibility and durability in undergraduate lab settings. Published case studies using simpler homemade systems in undergraduate laboratories may provide an alternative/complementary approach that has been applied in chemical engineering settings as reported by teams at Eindhoven/Amsterdam^104^ and Leeds.^105^

By recognizing the need for training toward a different skill set universities have started training chemistry undergraduate students through flow chemistry lectures and laboratories, which are vital steps in allowing a roll-out of such activities. With financial and logistical support from funding agencies and industry, academic leaders in this area can be empowered to expand their teaching efforts. SSPC’s role to bring together leaders from industry and academia in Ireland has already created a Community of Practice on the topic of flow chemistry in 2022/23 that aims to share knowledge and fertilize future efforts in this area that will go beyond individual collaborations between academic groups and industry partners.^119^

Ireland over the past 20 years has taken a number of innovative steps to ensure that the skills required to underpin the ongoing development and evolution of the pharmaceutical sector are available in a timely manner to match the growth in skills demanded. For example, NIBRT^106^ was developed to provide a mechanism to ensure the delivery of skills in bioprocessing and biopharmaceuticals through leveraging complementary input from the industrial development agency, leading companies, and academic institutions. An equally timely initiative in a center of excellence at the interface of flow technology, machine learning, and automation to underpin future advances in API manufacturing is warranted to ensure Ireland continues to provide the skills to lead in these transdisciplinary areas.

## Conclusion

It is clear that substantive advances have been made in the transition from traditional batch API synthesis to the use of continuous flow processing, where appropriate, with clear benefits. Navigating this change within a highly regulated sector brings challenges and opportunities. Ireland’s pharmaceutical landscape consisting of a globally significant cluster of large multinational API sites, complemented by CDMOs, and with effective mechanisms to encourage academic–industry partnership provides an interesting overview of various aspects of this transition. Given the small size of the country and the good connectivity between industry, academia, and government agencies, networking and shared learning on noncompetitive aspects is a clear advantage, which facilitates development at a sector level. Continued advances can be anticipated over the coming years with, for example, increased use of machine learning and automation.

## Notes

We dedicate this publication to Prof. Steven V. Ley—a pioneer in the field of continuous flow chemistry—on the occasion of his 80th birthday.
